# *Cryptosporidium* infection and associated risk factors among diabetic patients with diarrhea attending the Kosti Diabetic Centre (KDC) in Kosti City, White Nile State, Sudan

**DOI:** 10.1016/j.parepi.2026.e00564

**Published:** 2026-11-06

**Authors:** Abdelhakam G. Tamomh, Rabah M. Ibrahim, Nijoud A. Ali, Duaa H. Ahmed, Rayan G. Abdalla

**Affiliations:** Department of Parasitology and Medical Entomology, Faculty of Medical Laboratory Sciences, University of El Imam El Mahdi, Kosti 27711, Sudan

**Keywords:** *Cryptosporidium*, Diabetes mellitus, Opportunistic parasite, Diarrhea, Sudan

## Abstract

Cryptosporidiosis is an intestinal coccidian parasitic infection that in developing countries, results in diarrheal illness and death, particularly in immunocompromised persons. To date, published data on its prevalence among diabetic patients with diarrhea in Sudan are limited. Accordingly, this study aims to determine the prevalence of *Cryptosporidium* among diabetic patients with diarrhea attending to Kosti Diabetic Centre (KDC) in Kosti city, Sudan. A hospital-based cross-sectional study was employed among diabetic patients attending to KDC between 20 July 2022 and 30 January 2023. In total, 275 stool samples were collected and analysed using the modified Ziehl-Neelsen (ZN) staining method, thenexamined microscopically for *Cryptosporidium* infection. The overall prevalence of *Cryptosporidium* infection among diabetic patients was 80/275 (29.1%). A higher prevalence of 15/36 (41.6%) was found in diabetic patients with diarrhea compared with 65/239 (27.2%) in diabetic patients without diarrhea. Assessing the prevalence of *Cryptosporidium* infection among diabetic patients with diarrhea may help improve their health status, thereby supporting better health care and treatment and, consequently, improving the condition of diabetic patients.

## 1 Introduction

*Cryptosporidium* species rank among the most significant waterborne pathogens distributed worldwide (Abate et al., 2025; Mahamud et al., 2026; Razzolini et al., 2020). *Cryptosporidium* is sed as a primary etiological agent of diarrheal disease and a major cause of diarrheal disease in immunosuppressed persons. It is an opportunistic parasite that infects the gastrointestinal epithelium, producing potentially life-threatening diarrhea, especially in individuals with diabetes mellitus. Diarrhea in patients with immunocompromised conditions such as diabetes mellitus, HIV or patients on high doses of steroids is commonly caused by a *Cryptosporidium* parasite (Robinson et al., 2022; Mahamud et al., 2026; Shi et al., 2026).

*Cryptosporidium* infection may be transmitted through contamination of water and food with cryptosporidium oocysts (Bitilinyu-Bangoh et al., 2024; Tamomh et al., 2024, Tamomh et al., 2026). Cryptosporidiosis is a zoonotic disease, and severe symptoms can occur in immunocompromised persons, leading to severe diarrhea and abdominal cramps. The effects of diarrhea are easily observed in immunocompromised individuals and children, who may die from severe water loss (Sone et al., 2025; Tamomh et al., 2025).

A diarrheal disease caused by the parasite *Cryptosporidium*, has signigificant public health implication, and by extension, economic implications. The complications and economic burden of cryptosporidiosis are likely to be severe in diabetics than in healthy individuals (Didarlu et al., 2024; Semmani et al., 2023). To broaden the differential diagnosis for gastrointestinal manifestations beyond *Cryptosporidium*, healthcare professionals must consider a wide range of infectious agents, environmental influences, and toxic compounds that compromise intestinal barrier integrity (Sone et al., 2025). Gastrointestinal helminth infections are a major global contributor to diarrheal illness; mixed infestations involving nematode, trematode, and cestode species are common in livestock reservoirs, most notably sheep, and their close association with human agricultural settings serves as a primary conduit for contamination. Beyond biological macroparasites, contemporary toxicological agents pose a serious hazard to intestinal health. Specifically, metal-derived toxins from dietary or environmental sources, such as copper nanoparticles, has been shown to induce significant oxidative stress and cellular membrane instability, leading to direct injury to the intestinal epithelial lining (Casale et al., 2025). By down-regulating critical tight junction proteins and initiating robust inflammatory cytokine cascades, these cellular toxins can precipitate severe mucosal shedding, resulting in a mechanistic and malabsorptive diarrhea that may clinically resemble or exacerbate a parasitic infection (Luo et al., 2026). *Cryptosporidium* infections in low-and–middle income countries are cause a diarrheal illness associated with mortality in immunocompromised persons. Sudan, situated in Northeast Africa, is one of these countries facing economic and social crises. Some epidemiological surveys have demonstrated that infections resulting from food- and waterborne pathogens present a substantial public health challeng; *Schistosoma* spp., *Plasmodium* spp., and soil-transmitted helminths are endemic in Sudan (Sone et al., 2025; Tamomh et al., 2020, Tamomh et al., 2024, Tamomh et al., 2025, Tamomh et al., 2026). However, limited knowledge exists on the prevalence of *Cryptosporidium* infection among diabetic patients in Sudan. This study aims to determine the prevalence of *Cryptosporidium* infection among diabetic patients attending the KDC in Kosti City, White Nile State, Sudan.

## 2 Materials and methods

### 2.1 Study design, setting and population

A Cross-sectional study was conducted at Kosti Diabetic Centre (KDC) in Kosti City, White Nile State, Sudan, from July 20, 2022 to January 30, 2023. All patients attending at KDC during the data collection period were eligible for the study. Participants were recruited randomly, and written informed consent was obtained prior to participation in the study. KDC consults 65–130 diabetic patients a week (250–500 per month). Patients at the center include cases with type 2 diabetes, complicated type 1 pediatric diabetes patients and diabetic foot disease with complications. A team of internal medicine specialists, general and vascular surgeons, an ophthalmologist, a dental specialist, a nutritionist, and a podiatrist is available at KDC to offer care to such patients. The clinical laboratory is staffed by laboratory technicians who perform various investigations such as HbA1c, renal panel, and routine microscopy for pathogens. Although parasitologists at the main Kosti Teaching Hospital follow standard procedures for stool sample processing and examination, KDC laboratory technicians are trained to screen suspected diabetic patients for opportunistic pathogens such as *Cryptosporidium* spp. to protect immunologically weakened patients from severe wasting or malabsorption. This center serves patients from Kosti city and the surrounding localities.

### 2.2 Inclusion criteria

All diabetic patients receiving services at KDC during the study period were included. Patients presenting with or without diarrhea (diarrhea was defined as passing three or more loose or watery or semi-liquid stools over a 24 h period). Inclusion also required patientst to provided consent after the aims and procedures were thoroughly explained in either English or Arabic.

### 2.3 Exclusion criteria

Non-diabetic individuals, diabetic patients who declined to participate in the study, and patients diagnosed with serious systemic organ failure and pre-existing serious systemic diseases (such as gastrointestinal tract) were exempted. Also, patients who have recently received treatment with any anti-helminthic or anti-protozoal medication were not included in the study.

### 2.4 Ethical considerations

Ethical clearance for this study protocol was granted by the Institutional Ethics Committee of the Faculty of Medical Laboratory Sciences, University of El *Imam* El Mahdi (Date: January 6, 2022; Code No.: FMLS/UEE/EA/2022-3).

### 2.5 Data collection

A pilot test of the structured questionnaire was conducted to assess tool validity, sample feasibility, and laboratory processes before full-scale data collection. The collected data consisted of sociodemographic and clinic-related characteristics, including unique code number, age, gender, signs and symptoms, and place of residence.

### 2.6 Stool samples collection

Diabetic patients presenting with or without intestinal signs and symptoms were invited to submit a single stool sample in prelabeled clean stool containers. Provided by the research team Stool samples were collected three times a week in the morning (Sudanese local time). Patients were advised to avoid urine or water contamination while collecting the stool specimens About 30 g (about the size of a walnut) of formed stool, or 5 to 10 ml of watery or loose stool, were collected into the container, promptly sealed, and brought to the center the same day.

### 2.7 Sample handling and storage

Laboratory staff appropriately assessed the stool samples and recorded identified physical characteristics. Sample label on the containers was cross-checked with their corresponding questionnaires. Fresh stool samples were stored and preserved at −20 °C in the laboratory freezer at the end of each working day for subsequent batch analysis (Arora and Arora, 2026; Ionică et al., 2024; Tamomh et al., 2021a, Tamomh et al., 2021b).

### 2.8 Modified Ziehl Neelsen (mZN) staining technique

Fecal smears were prepared by spreading with wooden applicators on a clean glass slides and air-dried flat on racks. The smears were then fixed with pure methanol for 3 min, washed with water, then stained with strong carbol fuchsin for 15 min, washed under tap water again, then decolorized in 1% acid alcohol for 15 s, rinsed under tap water and stained with 1% methylene blue for 30 s. The oocysts were spherical to ovoid, measuring approximately 4.5–5.5 μm in diameter (Fig. 1). A three qualified microscopists examined the slides independently, and at least two agreeing reports were considered positive for C*ryptosporidium* (Arora and Arora, 2026; Tamomh et al., 2021a, Tamomh et al., 2021b).

**Fig. 1:**
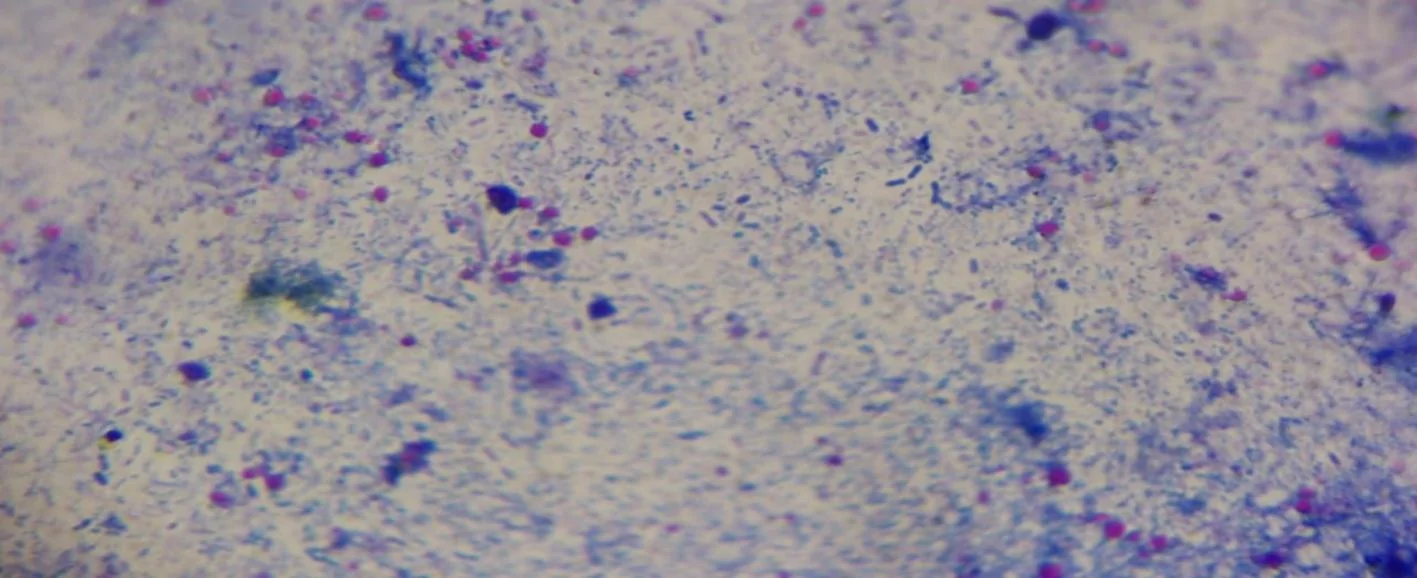
Oocyst of *Cryptosporidium.*

### 2.9 Statistical analyses

Data were recorded and entered into the Statistical Package for the Social Sciences (SPSS) Software, version 21, for analysis. Descriptive statistics were used to summarize the characteristics of the study population. Variables were summarized into frequencies, proportions, Odds Ratios (OR), and the corresponding 95% Confidence Intervals (CI). Chi-square and bivariate regression analyses were used to test the association between the dependent and independent categorical variables. *P* < 0.05 was considered statistically significant.

## 3 Results

### 3.1 Socio-demographic characteristics of study participants

A total of 275 diabetic patients participated in the study. Of the participants, 29.8% (82/275) were male and 70.2% (193/275) were female. Their mean age was 49.2 years with standard deviation, 13.4. Of all participants, 252 (91.6%), 16 (5.81%), and 7 (2.5%) were married, single, and widowed, respectively. By occupation, 22(8%), 9(3.3%), 12(4.4%), 164 (59.6%) and 68 (24.7%) were government employees, drivers, farmers, non-employed and others respectively. Based on their education levels, 164(59.6%), 9 (3.3%), 12 (4.4%), 68 (24.7%), 22 (8%), were illiterate (can't read or write), primary school(up to 9th grade), secondary school (up to 3rd grade), Khalwa (Quran school), and University (Bachelor's degrees to PhD level) respectively. According to the type of diabetes, 88 (32%) and 187 (68%) were type 1 and type 2 diabetes mellitus, respectively. Of all participants, 210 (76.4%) were rural residents and 65 (23.6%) were urban residents. Also, according to the duration of diabetes mellitus, 109 (39.6%), 88 (32%), 33 (12%), 26(9.5%), 12 (4.4%) and 7 (2.5%) had less than five years, six to ten years, eleven to fifteen years, sixteen to twenty years, twenty-one to twenty-five years and more than twenty-five years respectively as explained in (Table 1).

**Table 1:** Demographics data of the studied participants:

Demographics	Frequency	%
Age		
≤20	2	0.7
21–40	80	29.1
41–60	134	48.7
61–80	59	21.5
>80	0	0
Gender		
Male	82	29.8
Female	193	70.2
Marital status		
Married	252	91.6
Single	16	5.8
Widow	7	2.5
Occupation		
Government employee	22	8
Driver	9	3.3
Farmer	12	4.4
Other	68	24.7
Non-employed	164	59.6
Education level		
Illiterate	164	59.6
Primary school	9	3.3
Secondry school	12	4.4
Khalwa (Quran school)	68	24.7
University	22	8
DM type		
Type 1	88	32
Type2	187	68
Duration of DM		
<5 yrs	109	39.6
6-10 yrs	88	32
11-15 yrs	33	12
16-20 yrs	26	9.5
21-25 yrs	12	4.4
>25 yrs	7	2.5
Residence		
Urban	210	76.4
Rural	65	23.6

### 3.2 Prevalence of cryptosporidium and types and duration of diabetes mellitus

Among 275 study participants, the *Cryptosporidium* parasite was detected from 80 (29.1%) diabetic patients, and the remaining 195 (69.9%) were negative for *Cryptosporidium*. Of all participants examined by diabetes type, 88 (32%) and 187 (68%) had type 1 and type 2 diabetes mellitus, respectively; the parasite was detected in 26 (29.5%) and 54 (28.9%), respectively (OR = 1.033, 95% CI = 0.592–1.802, *P* = 0.909). From all participants examined according to the duration of diabetes mellitus, 109 (39.6%), 88 (32%), 33 (12%), 26(9.5%), 12 (4.4%) and 7 (2.5%) had less than five years, six to ten years, eleven to fifteen years, sixteen to twenty years, twenty-one to twenty-five years and more than twenty-five years respectively, and the parasite was detected in 34(31.2%), 28(31.8%), 5(15.2%), 7(26.9%), 3(25%), and 3(42.9%) respectively (*P* = 0.485) as shown in (Table 2)**.**

**Table 2:** Relationship between *Cryptosporidium* infection, the type of diabetes mellitus (DM) and the duration of diabetes mellitus.

DM type	No. examined	No. infected%	OR	95%CI	*P. value*
Type 1	88	26(29.5)			
Type2	187	54(28.9)	1.033	(0.592–1.802)	0.909
Duration of DM					
<5 yrs	109	34(31.2)	–	–	0.485
6-10 yrs	88	28(31.8)	–	–	
11-15 yrs	33	5(15.2)	–	–	
16-20 yrs	26	7(26.9)	–	–	
21-25 yrs	12	3(25)	–	–	
>25 yrs	7	3(42.8)	–	–	

### 3.3 Factors associated with cryptosporidium infection

In this study, individuals aged 21–40 years exhibited the highest infection rate (25, 31.3%), though this difference across age groups was not statistically significant (*p* = 0.774). Similarly, females had a higher prevalence of infection (61, 31.6%) than males (19, 23.2%), but this difference was not statistically significant (*p* = 0.159). Furthermore, no significant association was observed between *Cryptosporidium* infection and diabetic patients with different marital status (*p* = 0.174), occupations (*p* = 0.106), educational levels (*p* = 0.106), and residence (*p* = 0.513), as shown in (Table 3). Among the total of diabetic patients involved in this research, 110 had a habit of eating raw vegetables, and 24(30%) of them were infected with *Cryptosporidium*. There was a slightly significant association between eating raw vegetables and *Cryptosporidium* infection (OR = 0.543, 95% CI: 0.312–0.947, *p* = 0.030). The study showed that *Cryptosporidium* infection was significantly associated with contact to domestic animals ((OR = 0.543, 95% CI: 0.312–0.947, *p* = 0.030)), with approximately 46% lower odds of Cryptosporidium infection among those reporting contact than among those without such contact with domestic animals., manifestations of abdominal pain (OR = 1.938, 95% CI = 1.006–3.735, *p* = 0.046), vomiting (OR = 1.912, 95% CI = 0.930–3.933, *p* = 0.075), and diarrhea (OR = 3.872, 95% CI = 1.002–14.111, *p* = 0.028) as explained in (Table 4).

**Table 3:** Distribution of socio-demographic characteristics with *Cryptosporidium* infection among diabetic patients attending to Kosti Teaching Hospital, Sudan, 2022.

Characteristics	No. examined	No. infected%	OR	95% CI	*P-value*
Age					
≤20	2	0(0)	–	–	0.774
21–40	80	25(31.3)	–	–	
41–60	134	39(29.1)	–	–	
61–80	59	16(27.1)	–	–	
>80	0	0(0)	–	–	
Gender					
Male	82	19(23.2)	0.653	(0.472–1.144)	0.159
Female	193	61(31.6)	–	–	–
Marital status					
Married	252	73(28.9)	–	–	0.174
Single	16	3(13.8)	–	–	
Widow	7	4(57.1)	–	–	
Occupation					
Government employee	22	5(22.7)	–	–	0.106
Driver	9	2(22.2)	–	–	
Farmer	12	6(50)	–	–	
Other	68	13(19.1)	–	–	
Non-employee	164	54(32.9)	–	–	
Education level					
Illiterate	164	54(32.9)	–	–	0.106
Primary school	9	2(22.2)	–	–	
Secondary school	12	6(50)	–	–	
Khalwa(Quran school)	68	13(19.1)	–	–	
University	22	5(22.7)	–	–	
Residence					
Rural	210	59(73.8)	0.819	(0.449–1.493)	0.513
Urban	65	21(26.2)		.

**Table 4:** Risk factors associated with *Cryptosporidium* infection among diabetic patients attending to Kosti Teaching Hospital, Sudan, 2022.

Factors	No. examined	No. infected(%)	OR	95% CI	*P-value*
Eating raw vegetable					
Yes	110	24(30)	0.543	(0.312–0.947)	0.030
No	165	56(70)			
Contact with domestic animals					
Yes	110	24(30)	0.543	(0.312–0.947)	0.030
No	165	56(70)			
Abdominal pain					
Yes	46	19(23.7)	1.938	(1.006–3.735)	0.046
No	229	61(76.3)			
Vomiting					
Yes	36	15(18.8)	1.912	(0.930–3.933)	0.075
No	239	65(81.2)			
Diarrhea					
Yes	10	6(7.5)	3.872	(1.002–14.111)	0.028
No	265	74(92.5)			

## 4 Discussion

*Cryptosporidium* species are pathogens that cause diarrheal disease globally, and can be more severe in diabetic patients (Alemu et al., 2018; Azhary et al., 2025; Ngobeni et al., 2022; Taghipour et al., 2024; Semmani et al., 2023). Therefore, studying the prevalence of *Cryptosporidium* infection among diabetic patients contributes to establishing non-diabetic related complications such as *Cryptosporidium* induced diarrhea. It is against this backdrop that this study aimed to determine the prevalence of *Cryptosporidium* infection among diabetic patients with diarrhea attending the KDC. In our present findings, the overall prevalence of *Cryptosporidium* infection was 29.1% among the diabetic patients. Comparatively, the current prevlance was lower than that obtained in North West Ethiopia (42%), but higher than report from Southern Ethiopia, Egypt, Cameroon and Ghana;8.4%, 12%, 0.67%, and 15% respectively (Alemu et al., 2018; Sisu et al., 2021; Taghipour et al., 2024; Waly et al., 2021). These findings suggest that compromised immunity in diabetics, coupled with exposure to environmental factors, may mediate transmission and infection by *Cryptosporidium* spp. This result suggested that the diabetic patients acquired parasitic infections resulting from their low immunity and may due to awareness less safety habits. Additionally, variations in transmission levels across study areas, sanitary habits, health facilities, and environmental conditions may contribute to these difference. The intersection of *Cryptosporidium* infection and diabetes mellitus creates a perilous, bidirectional pathophysiological cycle in which diabetes increases susceptibility to the parasite, while subsequent infection profoundly compromises glycemic control (El-Nady et al., 2021). Chronic hyperglycemia precipitates secondary cellular immunodeficiency, undermining CD4+ T-cell responses and interferon-gamma production, both essential for parasite clearance. Concurrently, diabetic autonomic neuropathy impairs intestinal motility, thereby obstructing the mechanical expulsion of pathogens, whilst elevated blood glucose levels damage the tight junctions of the intestinal mucosal barrier (Ge et al., 2023; Balendran et al., 2024). This confluence facilitates even small numers of *Cryptosporidium* oocysts to readily adhere to, colonize, and devastate the microvilli of the small intestine, thereby transforming an opportunistic pathogen into a significant clinical peril for individuals with diabetes. Once established, *Cryptosporidium* exacerbates the patient's diabetes by inducing villous atrophy, which impairs brush-border enzymes and causes erratic, unpredictable nutrient absorption. Such malabsorption places patients at an elevated risk for sudden, life-threatening hypoglycemic episodes, as administered insulin may significantly overestimate the actual glucose influx into the bloodstream. Furthermore, the localized intestinal damage incites a systemic inflammatory response, thereby upregulating the production of pro-inflammatory cytokines, specifically TNF-α and IL-6, which directly exacerbate peripheral insulin resistance. In the framework of Type 1 diabetes, this infectious stress and the resultant glycemic variations can rapidly precipitate diabetic ketoacidosis (DKA), whereas in older individuals with Type 2 diabetes, the substantial depletion of diarrheal fluids results in acute dehydration, consequently concentrating blood glucose levels and posing a risk of inducing a hyperosmolar hyperglycemic state in conjunction with deteriorating renal function (Sohail et al., 2022).

Our current investigation revealed that female participants had higher infection rates, (31.6%) with *Cryptosporidium* as compared to male participants (23.2%). However, the difference was not statistically significant (*p* = 0.159) probably due to more domestic activities of females, especially in rural areas which might increase the chances of exposure to the infection.

The present study showed that most *Cryptosporidium* infection were detected among participants aged 41–60 years (29.1%). This could be due to declining immune system and may result from the long-term duration of diabetes (Taghipour et al., 2024).

Participants from rural areas (73.8%) were more infected with *Cryptosporidium* than those from urban areas (26.2%). This may be due to poor sanitation, unavailability of safe drinking water and limited awareness of control diabetes control. On the other hand, urban populations have access to clean water and they can visit diabetic clinics regularly for checkups.

The magnitude of *Cryptosporidium* infection among married individuals was 73(28.9%), but the difference was not significant (*p* = 0.174). Our study reported 32.9% *Cryptosporidium* infections among diabetic patients who were illiterates compared to the other levels of education. Poosibly attributable to ignorance of the disease and limited knowledge of the disease acquisition.

Our current study revealed that the *Cryptosporidium* infection was more among type 2 diabetic patients than type 1 diabetic patients, with insignificantly association (OR = 1.033, 95%CI = 0.592–1.802, *P* = 0.909). Additionally, the infection increase among who have had diabetes mellitus for more than 25 years (42.9%), with insignificantly association (*P* = 0.485).

From our investigated analysis the results showed that among the total of diabetic patients involved in this research there was a marginally significant association between *Cryptosporidium* infection and eating raw vegetables (OR = 0.543, 95% CI: 0.312–0.947, *p* = 0.030), and contact to domestic animals (OR = 0.543, 95% CI: 0.312–0.947, *p* = 0.030), with approximately 46% lower odds of Cryptosporidium infection. The present findings revealed that participants' occupation was not significantly associated with *Cryptosporidium* infection. However, there was a significantly association of *Cryptosporidium* infection among diabetic patients diarrhea (*p* = 0.028).

### 4.1 Limitation of the study

This study utilized a cross-sectional design and is carried out within a single-center framework at the Kosti Diabetic Center, thus constraining the ability to generalize the findings to the entire diabetic population in Sudan. Moreover, reliance on the modified Ziehl-Neelsen staining technique, rather than more advanced molecular methods such as polymerase chain reaction (PCR), may have reduced accurate estimation of true prevalence and limited identification of distinct *Cryptosporidium* species or genotypes involved in transmission dynamics. Freezing and thawing stool samples may cause oocyst undercounting and fragmentation. Additionally, due to the large volume of sample collection across different timelines and logistical constraints at the collection sites, immediate processing or continuous 4 °C storage was not feasible for all samples. Freezing at −20 °C was chosen to stop any potential protozoan or bacterial replication and preserve the structural integrity of the oocyst walls for subsequent batch processing.

## 5 Conclusion

In conclusion, these findings provide a new perspective on the epidemiology of *Cryptosporidium* infection in the global diabetic population and represents a first step in domestic data reporting. This study reveals a notable prevalence of *Cryptosporidium* infection (29.1%) among diabetic individuals presenting with diarrhea at the KDC, thereby highlighting a potentially hazardous bidirectional pathophysiological connection in which chronic hyperglycemia compromises intestinal immunity, while the parasite simultaneously disrupts glycemic control and triggers severe metabolic crises. The results reveal that factors such as rural residency, illiteracy, raw vegetable consumption, and the interaction with domestic animals are pivotal facilitators of transmission, emphasizing that females and individuals aged 41–60 bear the greatest disease burden. Ultimately, these findings highlight the critical necessity of incorporating routine parasitological screening within diabetic care protocols and formulating targeted public health interventions centered on enhancing access to clean water, promoting health and sanitation education, and ensuring safe animal handling practices to improve health outcomes for at-risk diabetic populations in Kosti city of the White Nile State in Sudan.

## CRediT authorship contribution statement

**Abdelhakam G. Tamomh:** Writing – review & editing, Writing – original draft, Visualization, Validation, Supervision, Software, Resources, Project administration, Methodology, Investigation, Funding acquisition, Formal analysis, Data curation, Conceptualization. **Rabah M. Ibrahim:** Writing – review & editing, Writing – original draft, Software, Resources, Investigation, Formal analysis, Data curation, Conceptualization. **Nijoud A. Ali:** Writing – review & editing, Software, Resources, Methodology, Investigation, Data curation, Conceptualization. **Duaa H. Ahmed:** Writing – review & editing, Software, Methodology, Investigation, Data curation, Conceptualization. **Rayan G. Abdalla:** Writing – review & editing, Software, Methodology, Investigation, Data curation, Conceptualization.

## Ethics approval and consent to participate

Institutional Ethics Committee approval was obtained from the Faculty of Medical Laboratory Sciences, University of El *Imam* El Mahdi (Date: January 6, 2022; Code No.: FMLS/UEE/EA/2022-3). Participants provided informed consent and received detailed information regarding their rights and duties prior to their involvement in the research. The experimental procedures were performed in accordance with the applicable rules, regulations, and the principles laid out in the Declaration of Helsinki.

## Funding

None.

## Declaration of competing interest

All the authors declared that they did not have conflict of interest.

## Data Availability

The data used to support the findings of this study are available from the corresponding author upon request.
